# Introduction of the unplanned extubations bundle in a tertiary cardiothoracic critical care unit: does it make any difference?

**DOI:** 10.1186/2197-425X-3-S1-A938

**Published:** 2015-10-01

**Authors:** S Gelvez-Zapata, R D'Oliveiro, J Osgathorpe, J Lonsdale, M Petty, N Jones

**Affiliations:** Intensive Care, Papworth Hospital NHS Foundation Trust, Cambridge, United Kingdom

## Introduction

Unplanned extubation (UE) is a major complication of endotracheal intubation in patients with mechanical ventilation, occurring in 3% to 16% of cases and an indicator of quality of care. However, the risk factors and downstream impacts of UE are poorly understood.

## Objectives

To evaluate the incidence and risk factors associated with UE in our Intensive Care Unit (ICU) and, based on these findings, develop a new strategy to decrease the number of UE events and improve clinical outcomes.

## Methods

This review looked at UE over a 24-month period (1^st^ April 2013 - 31^st^ March 2015) in the ICU of a tertiary cardiothoracic centre. We investigated the factors the contributed to UE over an initial period of 13 months. Literature review and consultation with the colleagues involved in the care of the patients in ICU were used to implement a new strategy to decrease UE. Further data were collected following the implementation of this strategy.

## Results

**Figure 1:**
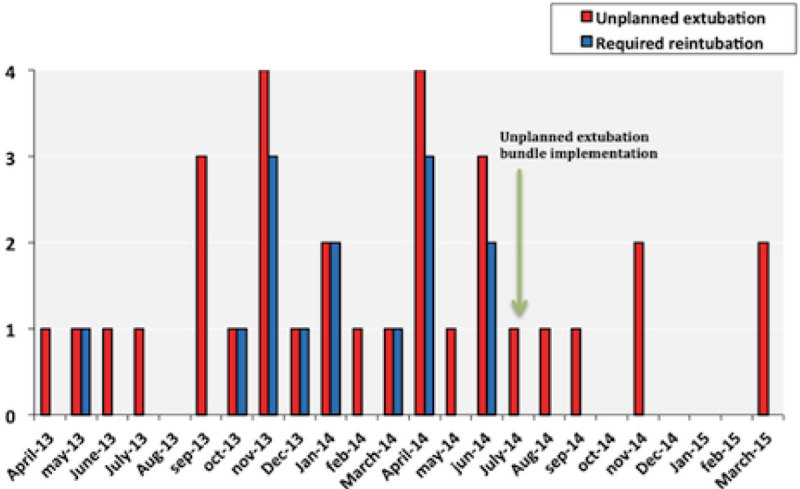
**Incidence of unplanned extubations before and after the implementaion of the care bundle**.

32 UE events were detected, with 14 patients requiring reintubation over the 24-month data collection period. Initial rates were low, but increased from November 2013 and persisted at a high rate for 6 months. The first results were analysed after 13 months of data collection. 21 UE and 12 reintubations were found. The most common causes found were that the endotracheal tube (ETT) had been cut too short, the patient was restless or agitated, no direct supervision when the patient self-extubated, sedation being off at the time of self-extubation or the sedation line being periodically occluded. Literature review did not suggest that one type ETT tie or tying technique was preferable, so we continued with ribbon gauze and soft (blue) ties and the practitioner's preferred tying method. Based on our findings, an Unplanned Extubation Bundle was launched in July 2014 focussing on 3 major points: ETT tube care (appropriate length, secure fixation, regular position checks and rapid response to displacement), sedation (appropriate choice and depth), and spontaneous awakening and breathing trials every day. Additionally, study days have been implemented to improve staff training. New data collected between July 2014 and March 2015 showed just 7 UE with no reintubations since the Unplanned Extubation Bundle was implemented (Figure 1). Following the introduction of the Bundle, we observed a 53% reduction in the overall rate of UE (p < 0.037, Poisson test). We also observed a reduction in the rate at which patients required reintubation following UE, with no reintubations required in any of the 7 UE events that occurred following the introduction of the Bundle (p < 0.039, Poisson test).

## Conclusions

Implementation of an Unplanned Extubation bundle was associated with a significant reduction in UE with no need for reintubation.
